# Repercussions of the COVID-19 Response in Pregnant Women in Western Uganda: Knowledge, Behavior, and Emotional State after the First Lockdown in 2020

**DOI:** 10.3390/ijerph18157817

**Published:** 2021-07-23

**Authors:** Stefanie Theuring, Agnes Kengonzi, Lorena Hafermann, Carolin Herrmann, Steven Ndugwa Kabwama, John Rubaihayo

**Affiliations:** 1 Institute of Tropical Medicine and International Health, Charité-Universitätsmedizin, Corporate Member of Freie Universität Berlin, Humboldt-Universität zu Berlin, and Berlin Institute of Health, 13353 Berlin, Germany; 2 School of Health Sciences, Mountains of the Moon University, Fort Portal P.O. Box 837, Uganda; kenaggie24@gmail.com (A.K.); rubaihayoj@yahoo.co.uk (J.R.); 3 Institute of Biometry and Clinical Epidemiology, Charité-Universitätsmedizin, Corporate Member of Freie Universität Berlin, Humboldt-Universität zu Berlin, and Berlin Institute of Health, 10117 Berlin, Germany; lorena.hafermann@charite.de (L.H.); carolin.herrmann@charite.de (C.H.); 4 School of Public Health, Makerere University, Kampala 7062, Uganda; skabwama@musph.ac.ug

**Keywords:** Uganda, COVID-19, KAP study, knowledge, prevention behavior, emotional stress, pregnant women

## Abstract

Limited research exists on pregnant women’s knowledge, attitudes, and behavior concerning COVID-19 in sub-Saharan Africa. We performed a cross-sectional study among 648 pregnant women in Fort Portal, Uganda, after the first lockdown starting in June 2020. Structured interviews were conducted at three different facilities during routine antenatal care, assessing sociodemographic background, knowledge of COVID-19, prevention behavior adherence, and psycho-emotional stress levels. We performed descriptive analyses and examined associated factors using multivariable logistic regression. In Fort Portal Region, 32.8% of pregnant women had a higher knowledge regarding the COVID-19 pandemic, while all women at least heard of COVID-19. 88.6% of the women showed low self-reported prevention behavior adherence. More than one third of the pregnant women experienced high psycho-emotional stress related to the pandemic (39.8%). The odds for psycho-emotional stress were increased among the age group 21–30 years (AOR 1.97; 95% CI 1.18–3.35) compared to women under the age of 21, and decreased in single or divorced women compared to women in partnerships (AOR 0.42; 0.22–0.77) and in women having less COVID-19-related knowledge (AOR 0.40; 0.27–0.58). In conclusion, prevention behavior adherence seemed challenging, and psycho-emotional stress was ubiquitous among our cohort. To avoid adverse consequences in maternal and neonatal health, campaigns for hygiene but also women’s emotional state should be a major focus of community healthcare in exceptional times such as the SARS-CoV-2 pandemic.

## 1. Introduction

In early 2020, when the World Health Organization (WHO) declared the emerging respiratory infection Coronavirus disease 2019 (COVID-19) to be a public health emergency of international concern [1], one of the greatest fears of the international health community was the spread of the virus to low- and middle- income countries with inadequate and often non-functional health systems [2]. Despite the fact that compared to other regions of the world, most African countries were initially spared the main burden of the pandemic [3], case numbers are on the rise, with the number of confirmed COVID-19 cases exceeding 42,000 in Uganda as of May 2021 [4]. While approximately 15% of COVID-19 patients will experience severe disease requiring oxygen support, and 5% will develop critical disease [5], most sub-Saharan African countries are highly unprepared for a large number of patients. Uganda has less than one hospital bed per 1000 people and only 137 intensive care unit beds in public hospitals for 46 million inhabitants [6]. Consequently, Uganda enforced strict measures to prevent COVID-19, implementing a nationwide lockdown five days before the first case of COVID-19 was detected [2,7]. As reported from a presidential address on 18 March 2020, all public gatherings, institutions, and events were suspended for around two months [7,8]. Apart from the strict lockdown, the Ugandan National COVID-19 strategy [6] also emphasized raising public awareness on risk factors for transmission and promoting infection prevention and control practices to mitigate the spread of COVID-19. Yet, the adherence to rigid infection control and prevention measures is often challenging in resource-limited settings. People in poorer communities often live together in cramped housing conditions with poor access to clean water, making social distancing or frequent handwashing extremely difficult [3].

So far, there is limited insight into knowledge, awareness, and practices (KAP) within sub-Saharan African communities with regard to COVID-19 and applied response strategies. In Uganda, the few existing KAP studies have targeted health workers [9] or medical students [10] and/or were conducted through online assessments [11,12], thus potentially biasing outcomes by neglecting poorer or less educated parts of the population, while mostly featuring only small samples. KAP studies assessing the situation of pregnant women, even though being particularly vulnerable during public health emergencies such as the SARS-CoV-2 pandemic, are even scarcer [13,14]. Pregnant women are prone to respiratory pathogens and the development of severe pneumonia due to altered cell-mediated immunity and pulmonary function, and viral pneumonia is assumed to be among the most frequent non-obstetric infectious diseases during pregnancy [15,16]. Hence, pregnant women might be more susceptible to COVID-19 than the general population [15]. Apart from a physical risk for maternal health, it has also been suggested that increased psychological stress related to the pandemic might harm pregnant women´s health and wellbeing and could also compromise neonatal and infant health by leading to intrauterine growth restrictions or neurodevelopmental disorders [17]. Accordingly, pregnant women require categorization as the key at-risk population in measures focusing on the prevention of SARS-CoV-2 infection [15]. In April 2020, the Uganda Ministry of Health published guidelines for care during pregnancy, delivery, and postnatal care in the context of COVID-19. The guidelines established strategies to mitigate the impact of the pandemic on pregnant women, such as conducting targeted antenatal care (ANC) outreaches, continuation of provision of family planning services, screening pregnant women for intimate partner violence, and screening pregnant women for signs and symptoms of COVID-19, among others [18]. However, there is no evidence if and how these guidelines are implemented in practice.

The goal of our study was to gain insight into knowledge, prevention behavior adherence, generally adapted behavior patterns, and psycho-emotional stress levels in the context of SARS-CoV-2 among pregnant women who experienced lockdown in Fort Portal, Uganda.

## 2. Materials and Methods

We conducted a cross-sectional KAP study among pregnant women accessing ANC services in Fort Portal, Western Uganda. Fort Portal municipality, located in Kabarole District, has a population of about 50,000; 98% of the households live within 5 km to the nearest health facility. There are about 48% women of reproductive age and about 5% expected pregnancies per year from the total Ugandan population [19]. The governmental recommendation for Ugandan women is to have four ANC visits during their pregnancy; about 40% of women are covered in the fourth visit [19]. The study sites comprised three health facilities: a private catholic hospital (Virika) and a public referral hospital (Buhinga), both located in Fort Portal town, and a governmental health center (Kibiito) in the rural surroundings of Fort Portal.

Women were recruited for a larger study on HIV prevention counselling when approaching ANC for the first time in their current pregnancy. Due to the eligibility criteria of the larger study, the cohort did not include HIV-positive women and women in a gestational age above 28 weeks. We included pregnant minors if they were 14 and above (“emancipated minors” [20]) due to their specific relevance regarding vulnerability. Our present study on COVID-19 was conducted among a subset of participants of this larger HIV prevention study. Recruitment and data collection started directly after the first lockdown phase in Uganda in June 2020. For our subset, up to September 2020, we included all women who were recruited for the overarching HIV prevention study, resulting in a sample of 648. Participants were interviewed after their routine ANC visit by trained local study nurses capable of local languages using a structured questionnaire.

Next to sociodemographic information, we assessed economic status by household assets, including radio, fridge, motorbike or car, electricity, tap water, cupboard, TV, cattle, and mosquito net. Each item was given one point upon presence, resulting in a wealth score ranging from 0 to 9 [21,22]. The questionnaire further contained a section on COVID-19, which had been adapted from the WHO-suggested approach to behavioral insights research for COVID-19 [23]. Questions covered knowledge, adherence to preventive behavior, and psycho-emotional stress. The questionnaire also covered general behavioral adaptations in the wake of the pandemic. Scale ends of questions and phrasing of questions are visible in the respective tables (also see Supplementary Material, Table S1 and Table S2).

We created binary relative categories (“higher” versus “lower”) for our outcome variables due to the lack of validated scores in the context of COVID-19 at the time of data collection. A higher knowledge status on COVID-19 was defined as: knowing the common symptoms “fever”, “cough”, “shortness of breath”, and the specific symptom “loss of taste and smell” AND knowing the incubation period of 14 days AND knowing that there was (at time of study conduction) no vaccine or treatment for COVID-19. Higher prevention behavior adherence was defined as the person stating to practice all of the following “often”: washing their hands, wearing a facemask, using disinfectants, avoid touching their face, keeping 2 m distance from others, and staying at home in case of symptoms. These items were chosen because they represent the most commonly propagated measures. A higher level of psycho-emotional distress was defined as worrying “a lot” (as opposed to “to some extent/not at all”) about all of the following items: own physical health, pregnancy being affected, losing someone beloved, and being unable to pay bills. We combined those items because they represent a mental burden in four different major areas of life in pregnant women (i.e., general health, pregnancy, family, and finances).

The underlying sample size of 648 pregnant women is solely based on the above-described subset of participants in the larger HIV prevention study from June until September 2020, so that no formal sample size calculation was conducted in advance. Therefore, all following calculations are to be understood descriptively. Categorical outcomes are described by absolute and relative numbers; ordinal and metric outcomes are presented by median and range or mean and standard deviation, respectively (Table 1). Factors associated with a lower knowledge level about COVID-19, influencing factors on lower prevention behavior adherence and factors associated with higher psycho-emotional stress level were additionally reported by odds ratios (OR) and 95% confidence intervals (95%CI) as well as adjusted odds ratios (AOR) with 95%CI. Reference groups, as well as covariables for the adjusted models, are shown in the respective Table 2, Table 3 and Table 4. Confidence intervals were not adjusted for multiple testing, and p-values are not reported due to the exploratory study design. The numbers of missing values per analysis were relatively low, so no imputation was conducted (explicit numbers of missing values are reported in the corresponding tables). The calculations were performed with R version 3.6.1, and the package “oddsratio” was used.

## 3. Results

We interviewed 648 pregnant women who visited one of the three participating health facilities for their first ANC visit. Sociodemographic characteristics are displayed in Table 1. Key baseline characteristics of our sample, such as age, marital status, education, or occupation, were largely corresponding with another sample of pregnant women in Western Uganda [22], implying comparability of our sample for pregnant women in this region.

### 3.1. Knowledge of SARS-CoV-2

All participants heard of SARS-CoV-2 in some form, mostly by radio, TV, or through other people in the community. Symptoms of fever (603; 93.2%), cough (605; 93.4%), and rhinorrhea (588; 91.0%) were most commonly known, followed by shortness of breath (581; 89.7%), headache (507: 78.5%), and sore throat (504; 77.8%). Fewer women knew that loss of taste and smell (331; 51.2%) or diarrhea (152; 23.5%) could also be the symptoms. 545 (84.2%) of the women correctly stated the incubation period of 14 days (Supplementary Material Table S1, Figure S1).

According to our definition, 208 (32.8%) participants showed a higher knowledge of COVID-19, while 427 (67.2%) had a lower knowledge level. The clientele at the private Virika hospital displayed lower knowledge compared to the public Buhinga hospital. Women who were single or divorced tended to have lower knowledge than coupled women in multivariable analysis (AOR 1.74; 1.01–3.07). Within this model, we could not identify other factors linked with knowledge level (Table 2).

### 3.2. Hygiene Behavior and General Behavioral Adaptations

Wearing facemasks, coughing/sneezing etiquette, and washing hands were practiced “often” by 60.3% (391), 55.4% (358), and 55.9% (361), respectively. Only 225 out of 648 women (34.7%) reported to often keep 2 m physical distance to others. (Supplementary Material, Table S1) Altogether, 72 (11.4%) women fulfilled our definition of higher adherence to hygiene and preventive behavior, while 562 (88.6%) showed lower adherence (Table 3). Compared to the age group <21 years, women between the age of 21 and 30 years and between 31 and 40 years were less likely to show lower adherence to prevention behavior, similar to women with a higher wealth score compared to women with a lower wealth score. The only difference with a stable association after adjusting was the attended health institution, with clients at the private (Virika) and the rural (Kibiito) health institutions showing lower odds of lower adherence compared to clients of the rural public Buhinga hospital.

As to COVID-19 related behavioral adaptations, 92.4% (599) stated they were avoiding people from other geographic regions like China or Europe, 89.7% (581) bought personal protective equipment, 83.5% (540) stocked up their homes with food supplies, 51.9% (334) reported that they had asked family and friends to refrain from visiting, and 48.4% (313) exercised less than before. The majority stated it would be unlikely that due to the pandemic, they would eat unhealthier food or drink more alcohol. Just 13.5% (87 women) stated they had difficulties receiving healthcare or ANC services. (Supplementary Material Table S2)

### 3.3. Psycho-Emotional Stress

**Figure 1:**
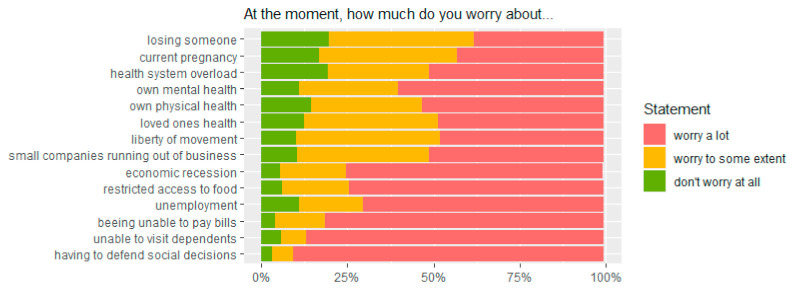
Statements on psycho-emotional stress during the pandemic.

The extent of various emotional concerns is summarized in Figure 1 and Supplementary Material Table S2.

We identified 251 (39.8%) women with a higher overall psycho-emotional stress level as per our definition. The age groups 21–30 and 31–40 years had increased odds for higher psycho-emotional stress as compared to women below 21 years. This could be confirmed for the age group 21–30 years compared to <21 years in multivariable analysis (AOR 1.97, 1.18–3.35). (Table 4) Being single or divorced was associated with lower odds for psycho-emotional stress than being married or coupled (AOR 0.42; 0.22–0.77). The odds for higher psycho-emotional stress increased with educational level: compared to women with no education, women with tertiary education had a six-fold increased risk for higher psycho-emotional stress (AOR 6.80; 2.04–25.80). Women with two or more children were less likely to experience a high level of psycho-emotional stress (AOR 0.64; 0.42–0.95). Women with a low level of COVID-19 related knowledge were less likely to have a high emotional workload (AOR 0.40; 0.27–0.58). In women who were clients at Virika hospital, the risk for higher psycho-emotional stress was about twice as high as in Buhinga (AOR 2.82; 1.73–4.65) (Table 4).

## 4. Discussion

Our study represents one of the first large-scale assessments of pregnant women’s knowledge, behavior, and emotional wellbeing in the context of SARS-CoV-2 and the COVID-19 response in sub-Saharan Africa in 2020. In summary, we showed that in Fort Portal Region, pregnant women from all different socioeconomic backgrounds were overall informed and knowledgeable about the COVID-19 pandemic. However, self-reported compliance with hygiene behavior measures was relatively low, and a majority of the pregnant women was clearly burdened by worries and fears related to the pandemic.

One in three women demonstrated a higher knowledge level on SARS-CoV-2. The majority of the participants mentioned radio as their main information source. Throughout different health contexts in sub-Saharan Africa, the use of radio-based information promoted by official sources, reinforcing the region’s narrative tradition, has proven to be a highly accessible and effective medium for health information [24]. In our study population, knowledge was not dependent on education, occupation, and wealth status, which is encouraging in terms of low-threshold accessibility and quality of information campaigns on a local level.

While our cohort was reasonably adherent to single hygiene measures such as mask wearing or hand washing, only one in three women kept a physical distance of 2 m most of the time. This measure might be plainly impossible to apply in densely populated conditions, when working in informal sectors, or depending on local markets for alimentation. Juxtaposing official hygiene recommendations with their daily practicability in a particular setting is an important part of local public health decision making, as also pointed out in studies from Nigeria, South Africa, and India [25,26,27]. In this context, the reflexive adoption of Western countries’ COVID-19 responses by African leaders can be viewed as counter-productive, and measures like physical distancing might appear elitist and unrealistic in many resource-limited settings [27,28]. Wasdani and Prasad (2020) particularly note the economic implications involved in the ability to adhere to such measures, suggesting respective governmental support like interim subsistence allowances for women in informal jobs where physical distancing is impossible. This might be especially important for women, who are rarely formally employed in such settings.

Only one in ten women complied with our definition of higher behavior adherence most of the time. Clients from the urban public Buhinga hospital were less likely to show prevention behavior adherence compared to the private hospital and the rural public facility. The latter is in contradiction with other studies, where rural populations were significantly less compliant with COVID-19-related behavior changes [28,29]. We suspect that facility size might also play a role, with smaller rural health centers possibly featuring stronger personal client–staff connections leading to higher client commitment compared to large urban referral institutions, where services tend to be more impersonal, and client–staff communication might be less beneficial [30]. At the same time, health institutions only represent one of many players in promoting hygiene measures and behaviors, and our finding might as well point to an underlying unidentified confounder with regard to the Buhinga clientele. The same applies to the finding that in the catholic hospital, clients were less knowledgeable but more prone to the higher psycho-emotional stress level. Either way, identifying clientele differences across varying facilities might be valuable, as targeting entire healthcare settings for hygiene or knowledge campaigns could entail pragmatic and economical implementation advantages compared to identifying individual clients in need.

We found a large degree of general behavior adaptation to the pandemic situation among pregnant women, including stocking up food and protective equipment or alteration of social habits. Yet alarmingly, misleading COVID-19 information might have spiraled up xenophobic tendencies with our sample reporting to avoid people who, for example, look Chinese, especially considering that in countries like Uganda, Chinese laborers are ubiquitous, and there have been tensions and propensities of “othering” in the past years [31]. It is of utmost importance, in Uganda as anywhere else, that such tendencies are counteracted with comprehensive public information campaigns, educating people about tangible risks for infection in their living environment rather than perpetuating xenophobic prejudices.

One in eight women did not receive healthcare or ANC at any point due to the pandemic. Although this means that the majority of women did not have problems receiving care, it still is a concerning rate given the importance of healthcare during pregnancy and the catastrophic impact obstetric service disruption can have on maternal and neonatal health [32].

A large part of the women reported psychosocial impact and worries, with 38% of the women showing a particularly high psycho-emotional stress level. Although comparability might be limited, this percentage exceeds pre-pandemic findings, such as a large meta-analysis of 52 studies which identified an overall pooled prevalence of 23% for self-reported anxiety symptoms in pregnancy [33], or a study in South Africa, where 16% of pregnant women showed a high stress level. [34] From the time of the pandemic, 21% of pregnant women in Wuhan, China, showed signs of anxiety [14]. The high psycho-emotional stress level in our cohort is worrisome, given that antenatal stress and anxiety have significant implications for obstetric outcomes and pediatric health impairment, including increased risk for premature birth, fetal growth restriction, and obstetric complications [33,34,35]. Women with tertiary education and women with higher knowledge of the pandemic were at increased risk for psycho-emotional stress in our setting, which was similar to findings from Italy [36]. This may point to a higher awareness of overall health and social risks through the pandemic in the better-educated and -informed part of the population. While providing realistic information on the pandemic is crucial, it should be kept in mind that it might also cause serious anxieties and that vulnerable groups of the population should not be left alone with distressing knowledge. Telephone hotlines or focal points where further advice can be obtained could be worthwhile options to compensate for individual worries arising in the wake of information campaigns.

Women in their twenties, women in a relationship or marriage, and women who were a first-time parent or a parent of only one child were also more likely to experience higher psycho-emotional stress levels. While primiparity has been associated with higher antenatal stress levels [35,37], the other factors are somewhat surprising, as there has been evidence that pregnant women in more precarious situations, i.e., being very young, single, or providing for several children, are more affected by psycho-emotional stress [35,38,39]. Yet, being in a partnership does not always equal receiving support, as pointed out by Bilszta et al., who found antenatal depression less prevalent in unpartnered mothers compared to mothers with unsupportive partners [40]. A large meta-analysis by Biaggi et al. [35] concluded that not only marital status should be considered, but also couple relationship quality, and against this background, being a single mother could be better for antenatal mental health than being in a difficult partnership.

The strength of our study is that it represents a comprehensive assessment of COVID-19 related repercussions among a large sample of pregnant women in a low-income country and that we did not limit the study population to women able to access an online questionnaire, avoiding an omnipresent selection bias in KAP research during the COVID-19 pandemic. As a limitation, we cannot exclude some degree of desirability or reporting bias concerning health-related behaviors. Questionnaires based on self-reported behavior are potentially prone to over- or under-reporting, and our results must be seen in light of this possible bias. However, the study nurses were trained to interview in a non-judgmental style and probe answers in order to avoid biased results as much as possible. Also, we used a self-adjusted questionnaire, which narrows possibilities for comparison with other settings. More specific questions pertaining to pregnancy-related fears could have been informative to develop support interventions. However, we believe that in light of lacking respective data, the broad range of our findings will contribute to better understanding pregnant women’s situation during an ongoing pandemic.

## 5. Conclusions

In conclusion, although a certain level of knowledge on the pandemic was rather widespread, pregnant women were often unable to comply with hygiene behavior measures in their daily lives, and fears and worries were ubiquitous. Prevention campaigns for COVID-19 should clearly relate to the everyday realities of women in a given regional setting, realistically considering which measures are practicable to apply and offering solutions for those that are not. This should include governmental subsistence support, particularly for women in informal sector jobs, to enable them to follow pandemic measures despite precarious living conditions. Apart from that, the emotional state of pregnant women should represent a focus of outreaching community healthcare in an exceptional time such as the SARS-CoV-2 pandemic to avoid adverse consequences in maternal and neonatal health.

## Figures and Tables

**Table 1:** Sociodemographic characteristics of the women and their partners.

Total *n* = 648	N(%)
Age (*n* = 647)	25.5 (5.8) (mean, standard deviation)
Marital Status (*n* = 648)	
Married Couple Single Divorced	525 (81.0) 29 (4.5) 91 (14.0) 3 (0.5)
Health Facility (*n* = 648)	
Buhinga (urban, public) Virika (urban, private) Kibiito (rural, public)	322 (49.7) 141 (21.8) 185 (28.6)
Completed Education (*n* = 646)	
None Primary Secondary Tertiary	25 (3.9) 305 (47.2) 232 (35.9) 84 (13.0)
Occupation (*n* = 639)	
Farmer Trader/self-employed (unspecified) Homemaker Hair dresser Teacher Tailor Other	246 (38.5) 122 (19.1) 118 (18.5) 27 (4.2) 20 (3.1) 20 (3.1) 86 (13.5)
Religion (*n* = 647)	
Christian Muslim Other	604 (93.4) 35 (5.4) 8 (1.2)
Number of children living in household (*n* = 644)	1 (0; 7) (median, range)
Wealth score [21,22]	
Lower (0–4) Higher (5–9)	287 (44.3) 361 (55.7)
Partner occupation (*n* = 648)	
Farmer Trader/self-employed (unspecified) Workmen (e.g., builder, engineer, mechanic) Driver (e.g., taxi, motorbike taxi, truck) Civil service, armed forces Other	172 (26.5) 133 (20.5) 106 (16.3) 88 (13.6) 51 (7.9) 98 (15.2)
Financially dependent on partner (*n* = 648)	
Completely For the most part For some part Not at all	226 (34.9) 222 (34.3) 183 (28.2) 17 (2.6)
Physical violence and/or other partner harassment? (*n* = 647)	
Yes No	59 (9.1) 588 (90.9)

**Table 2:** Factors associated with lower knowledge level calculated by logistic regression (*n* = 635).

Variable	Lower Knowledge Level *n* = 427 (67.24%) N (%)	OR (95% CI)	AOR (95% CI)
Age groups			
<21 21–30 31–40 >40	92 (69.70) 254 (67.02) 76 (65.55) 5 (62.50)	reference 0.88 (0.57; 1.35) 0.83 (0.48; 1.41) 0.73 (0.17; 3.67)	reference 0.85 (0.53; 1.35) 0.80 (0.45; 1.42) 0.76 (0.17; 3.99)
Marital status			
Married or couple Single or divorced	362 (66.06) 65 (74.71)	reference 1.52 (0.92; 2.60)	reference 1.74 (1.01; 3.07)
Education			
None Primary Secondary Tertiary	17 (68.00) 201 (66.56) 146 (64.89) 63 (75.90)	reference 0.94 (0.37; 2.18) 0.87 (0.34; 2.05) 1.48 (0.54; 3.88)	reference 0.74 (0.29; 1.78) 0.79 (0.29; 1.98) 1.24 (0.39; 3.76)
Occupation			
Not formally employed Formally employed	329 (68.12) 98 (64.47)	reference 0.85 (0.58; 1.25)	reference 0.62 (0.38; 1.00)
Wealth score			
0–4 5–9	189 (67.50) 238 (67.04)	reference 0.98 (0.70; 1.37)	reference 0.95 (0.64; 1.40)
Source of knowledge			
TV or Radio Social Media or Internet Community, other people Church or hospital	395 (66.16) 4 (100) 17 (85.00) 8 (88.89)	reference 4.09 (0.74; 76.16) 2.90 (0.96; 12.52) --	reference 4.12 (0.71; 78.03) 2.07 (0.65; 9.20) ---
Health Facility			
Buhinga (urban, public) Virika (urban, private) Kibiito (rural, public)	196 (62.82) 115 (82.14) 116 (63.39)	reference 2.72 (1.69; 4.52) 1.03 (0.70; 1.50)	reference 2.63 (1.57; 4.53) 1.02 (0.67; 1.56)

OR—odds ratio; AOR—Adjusted odds ratio with all variables included in this table as covariables; CI—Confidence interval.

**Table 3:** Factors associated with lower prevention behavior adherence calculated by logistic regression (*n* = 634).

Variable	Lower Prevention Behavior Adherence *n* = 562 (88.64%) N (%)	OR (95% CI)	AOR (95% CI)
Age groups			
<21 21–30 31–40 >40	125 (95.42) 331 (87.34) 99 (85.34) 7 (87.50)	reference 0.33 (0.12; 0.74) 0.28 (0.10; 0.70) 0.34 (0.05; 6.79)	0.50 (0.18; 1.21) 0.44 (0.15; 1.19) 0.56 (0.07; 11.92)
Marital status			
Married or couple Single, widow, divorced	481 (87.77) 81 (94.19)	reference 2.26 (0.97; 6.59)	1.58 (0.62; 4.88)
Education			
None Primary Secondary Tertiary	22 (88.00) 277 (92.03) 199 (88.44) 64 (77.11)	reference 1.57 (0.36; 4.98) 1.04 (0.24; 3.29) 0.46 (0.10; 1.52)	1.28 (0.27; 4.40) 0.83 (0.17; 3.12) 0.68 (0.12; 3.15)
Occupation			
Not formally employed Formally employed	431 (89.42) 131 (86.18)	reference 0.74 (0.43; 1.30)	0.92 (0.47; 1.89)
Wealth score [21,22]			
0–4 5–9	257 (92.11) 305 (85.02)	reference 0.52 (0.30; 0.88)	0.54 (0.28; 1.02)
COVID-19 knowledge			
Higher Lower	184 (88.89) 378 (88.52)	reference 0.96 (0.56; 1.61)	1.15 (0.64; 2.03)
Emotional stress level			
Higher Lower	214 (84.58) 348 (91.34)	reference 1.92 (1.17; 3.16)	1.19 (0.66; 2.12)
Health Facility			
Buhinga (urban, public) Virika (urban, private) Kibiito (rural, public)	299 (95.83) 107 (76.43) 156 (85.71)	reference 0.14 (0.07; 0.27) 0.26 (0.13; 0.51)	0.18 (0.08; 0.36) 0.17 (0.08; 0.36)

OR—odds ratio; AOR—Adjusted odds ratio with all variables included in this table as covariables; CI—Confidence interval.

**Table 4:** Factors associated with higher psycho-emotional stress level calculated by logistic regression (*n* = 630).

Variable	Higher Psycho-Emotional Stress Level *n* = 251 (39.84%) N (%)	OR (95% CI)	AOR (95% CI)
Age groups			
<21 21–30 31–40 > 40	30 (22.90) 177 (46.95) 42 (36.52) 2 (28.57)	reference 2.98 (1.91; 4.76) 1.94 (1.11; 3.40) 1.35 (0.19; 6.60)	1.97 (1.18; 3.35) 1.81 (0.92; 3.59) 2.05 (0.26; 11.78)
Marital status			
Married/couple Single/divorced	232 (42.65) 19 (22.09)	reference 0.38 (0.22; 0.64)	0.42 (0.22; 0.77)
Education			
None Primary Secondary Tertiary	5 (20.83) 87 (29.00) 96 (43.05) 63 (75.90)	reference 1.55 (0.60; 4.80) 2.87 (1.11; 8.91) 11.97 (4.22; 39.98)	1.67 (0.62; 5.34) 2.70 (0.95; 8.82) 6.80 (2.04; 25.80)
Occupation			
Not formally employed Formally employed	167 (34.79) 84 (56.00)	reference 2.39 (1.65; 3.47)	1.38 (0.83; 2.27)
Wealth score			
0–4 5–9	91 (32.73) 160 (45.45)	reference 1.71 (1.24; 2.38)	1.03 (0.68; 1.55)
Number of children living at home			
0–1 ≥2	153 (43.97) 98 (34.75)	reference 0.68 (0.49; 0.94)	0.64 (0.42; 0.95)
Financial dependence on partner			
Not at all For some part For the most part Completely	4 (26.67) 80 (45.71) 92 (42.40) 75 (33.63)	reference 2.32 (0.76; 8.61) 2.02 (0.67; 7.49) 1.39 (0.46; 5.16)	1.55 (0.44; 6.56) 1.49 (0.42; 6.32) 1.10 (0.31; 4.67)
Emotionally or physically threatened by partner			
No Yes	229 (40.03) 22 (37.93)	reference 0.92 (0.52; 1.58)	1.63 (0.85; 3.09)
COVID-19 Knowledge			
High Low	104 (50.00) 147 (34.83)	reference 0.54 (0.38; 0.75)	0.40 (0.27; 0.58)
Health Facility			
Buhinga (urban, public) Virika (urban, private) Kibiito (rural, public)	110 (35.60) 85 (60.71) 56 (30.94)	reference 2.80 (1.86; 4.24) 0.81 (0.55; 1.20)	2.82 (1.73; 4.65) 1.28 (0.81; 2.03)

OR—odds ratio; AOR—Adjusted odds ratio with all variables included in this table as covariables; CI—Confidence interval.

## Data Availability

The data presented in this study and the R code are available on request from the corresponding author. The data are not publicly available due to the possible identifiability of participants and data containing sensitive information, as this substudy was conducted in the frame of an HIV-related research project.

## Supplementary Materials

The following are available online at https://www.mdpi.com/article/10.3390/ijerph18157817/s1, Figure S1: SARS-CoV-2 related knowledge in pregnant women, Table S1: Descriptive overview of knowledge on COVID-19 and prevention measures, Table S2: Descriptive overview of behavioral aspects and psychosocial impact of the pandemic.
