# Supplementary material for: Repercussions of the COVID-19 Response in Pregnant Women in Western Uganda: Knowledge, Behavior, and Emotional State after the First Lockdown in 2020
Source: Int J Environ Res Public Health. 2021 Jul 23;18(15):7817. doi: 10.3390/ijerph18157817 (PMC8345751; doi:10.3390/ijerph18157817)
Supplement: Supplementary file 1 [file ijerph-18-07817-s001.zip › ijerph-1291908-supplementary.pdf]

Supplementary Material

Table S1. Descriptive Overview of Knowledge on COVID-19 and Prevention Measures n = 648.

	N (%)
Ever heard of Coronavirus/COVID-19 (n=648)	
Yes	648 (100)
No	0 (0)
Main sources of information (multiple answers)*	
(n = 648)	369 (56.9)
TV	572 (88.3)
Radio	288 (44.4)
Other people/community	52 (8.0)
Social Media/internet	41 (6.3)
Phone messages	35 (5.4)
Hospital	18 (2.8)
Church	51 (7.9)
Other	
Known symptoms (multiple answers) (n = 648)	
Cough	605 (93.4)
Fever	603 (93.1)
Runny or stuffy nose	588 (91.0)
Shortness of breath	581 (89.7)
Headaches	507 (78.5)
Sore throat	504 (77.8)
Muscle body aches	405 (62.7)
Fatigue	397 (61.4)
Loss of taste or smell (n = 647)	331 (51.2)
Diarrhea	152 (23.5)
Knows there is no drug or vaccine** (n = 648)	
Correct answer	568 (87.7)
No correct answer/don't know	80 (12.3)
Knows incubation period (n = 648)	
Correct answer: up to 14 days	545 (84.1)
No correct answer/don't know	102 (16.8)
Know how to protect myself from infection (n = 648)	
Not at all/To a small extent	163 (25.2)
To a large extent/Very much so	485 (74.8)
Avoiding an infection is (n = 648)	
Extremely difficult/Somewhat difficult	102 (15.7)
Somewhat easy/Extremely easy	546 (84.3)
I follow the recommendations (n = 648)	
Not at all/To a small extent	99 (15.3)
To a larger extent/Very much so	549 (84.7)
**Prevention measures taken since start of the epidemic**	
Hand washing (n = 646)	
Never/Rarely	60 (9.3)
Sometimes	225 (34.8)
Often	361 (55.9)
Avoiding touching own face (n = 648)	
Never/Rarely	137 (21.1)
Sometimes	275 (42.4)
Often	236 (36.4)

Use disinfectants (n = 648)	
Never/Rarely	251 (38.7)
Sometimes	188 (29.0)
Often	209 (32.3)
Staying home when sick (n = 648)	
Never/Rarely	88 (13.6)
Sometimes	242 (37.4)
Often	318 (49.1)
Covering when sneezing/coughing (n = 646)	
Never/Rarely	54 (8.4)
Sometimes	234 (36.2)
Often	358 (55.4)
Wearing face masks (n = 648)	
Never/Rarely	53 (8.2)
Sometimes	204 (31.5)
Often	391 (60.3)
Physical distancing 2m to others (n = 648)	
Never/Rarely	126 (19.4)
Sometimes	297 (45.8)
Often	225 (34.7)
Staying home (n = 648)	
Never/Rarely	107 (16.5)
Sometimes	282 (43.5)
Often	259 (40.0)
Disinfecting surfaces (n = 648)	
Never/Rarely	311 (48.1)
Sometimes	222 (34.3)
Often	114 (17.6)
Disinfecting mobile phones (n = 644)	
Never/Rarely	470 (73.0)
Sometimes	115 (17.9)
Often	59 (9.2)
Not seeing family and friends for me is… (n = 648)	
Impossible/Hard	261 (40.3)
Not very hard/No problem	387 (59.7)

* Question was asked as "How have you heard of Corona?" as an open-ended question; ** as of June-September 2020.

Table S2. Descriptive overview of behavioral aspects and psychosocial impact of the pandemic n = 648.

**Behavioral Adaptions**	N (%)
Bought food supplies (n = 647)	
I already did that/I plan to do that	540 (83.5)
I don't plan to do that	107 (16.5)
Avoided people from other countries like China or Europe (n = 648)	599 (92.4)
I already did that/ I plan to do that	49 (7. 6)
I don't plan to do that	
Exercised less (n = 647)	
I already did that/ Likely I'll do that	313 (48.4)
Unlikely I'll do that	334 (51.6)
Drank more alcohol (n = 648)	
I already did that/Likely I'll do that	72 (11.1)
Unlikely I'll do that	576 (88.9)

Ate more unhealthy (n = 648)	
I already did that/ Likely I'll do that	145 (22.4)
Unlikely I'll do that	503 (77.6)
Asked family/friends not to visit (n = 644)	
I already did that/I plan to do that	334 (51.9)
I don't plan to do that	310 (48.1)
Bought PPE (n = 648)	
I already did that/I plan to do that	581 (89.7)
I don't plan to do that	67 (10.3)
Avoided going to health facility (n = 647)	
I already did that/I plan to do that	40 (6.2)
I don't plan to do that	607 (93.8)
**Pandemic affecting pregnancy care**	
Could not access healthcare/ANC, or was denied care (n = 644)<br>Changed facility/plans where to deliver the baby (n = 645)<br>Lacking access to information or medical supplies (n = 647)	87 (13.5)<br>8 (1.2)<br>8 (1.2)
**Currently having worries about…**	
Losing someone beloved (n = 648)	
worrying a lot	588 (90.7)
to some extent/ not at all	60 (9.3)
Current pregnancy (n = 648)	
worrying a lot	565 (87.2)
to some extent/ not at all	83 (12.8)
Health system overload (n = 648)	
worrying a lot	528 (81.5)
to some extent/ not at all	120 (18.5)
Own mental health (n = 648)	
worrying a lot	456 (70.4)
to some extent/ not at all	192 (296)
Own physical health (n = 648)	
worrying a lot	480 (74.2)
to some extent/ not at all	167 (25.8)
Liberty of movement (n = 647)	
worrying a lot	329 (50.9)
to some extent/ not at all	318 (49.1)
Economic recession (n = 647)	
worrying a lot	313 (48.4)
to some extent/ not at all	334 (51.6)
Restricted access to food (n=647)	
worrying a lot	342 (52.9)
to some extent/ not at all	305 (47.1)
Unable to pay bills (n = 648)	
worrying a lot	332 (51.2)
to some extent/ not at all	316 (48.8)
Unable to visit dependents (n = 648)	
worrying a lot	277 (42.7)
to some extent/ not at all	371 (57.3)
Having to defend social decisions (n = 648)	
worrying a lot	246 (38.0)

to some extent/ not at all	402 (62.0)
Unemployment (n = 648)	
worrying a lot	389 (60.0)
to some extent/ not at all	259 (40.0)
Small companies running out of business (n = 648)	
worrying a lot	310 (47.8)
to some extent/ not at all	338 (52.2)
Loved ones health (n = 646)	
worrying a lot	486 (75.2)
to some extent/ not at all	160 (24.8)

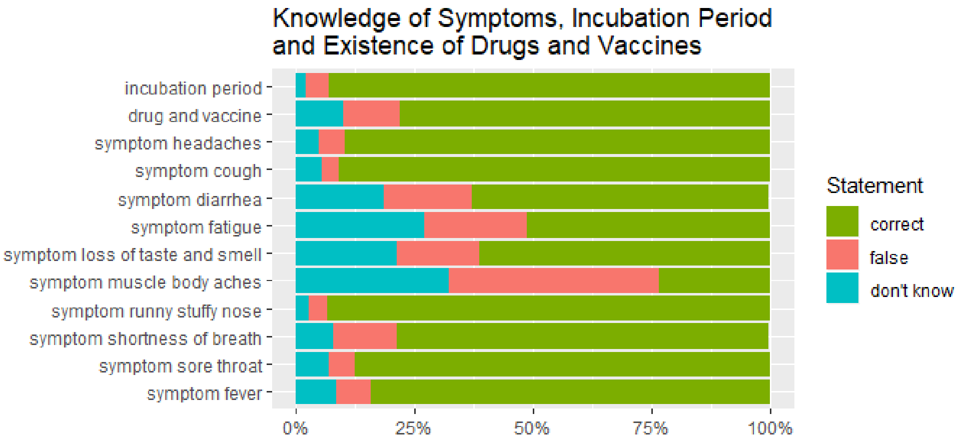

Figure S1: SARS-CoV-2 related knowledge in pregnant women.
